# Astrocytic modulation of cortical oscillations

**DOI:** 10.1038/s41598-018-30003-w

**Published:** 2018-08-01

**Authors:** Alba Bellot-Saez, Greg Cohen, André van Schaik, Lezanne Ooi, John W Morley, Yossi Buskila

**Affiliations:** 1 Biomedical Engineering and Neuroscience group, The MARCS Institute, Western Sydney University, Penrith, NSW Australia; 2 School of Medicine, Western Sydney University, Campbelltown, NSW Australia; 3 Illawarra Health and Medical Research Institute, School of Biological Sciences, University of Wollongong, Wollongong, NSW Australia

## Abstract

Brain waves are rhythmic voltage oscillations emerging from the synchronization of individual neurons into a neuronal network. These oscillations range from slow to fast fluctuations, and are classified by power and frequency band, with different frequency bands being associated with specific behaviours. It has been postulated that at least ten distinct mechanisms are required to cover the frequency range of neural oscillations, however the mechanisms that gear the transition between distinct oscillatory frequencies are unknown. In this study, we have used electrophysiological recordings to explore the involvement of astrocytic K^+^ clearance processes in modulating neural oscillations at both network and cellular levels. Our results indicate that impairment of astrocytic K^+^ clearance capabilities, either through blockade of K^+^ uptake or astrocytic connectivity, enhance network excitability and form high power network oscillations over a wide range of frequencies. At the cellular level, local increases in extracellular K^+^ results in modulation of the oscillatory behaviour of individual neurons, which underlies the network behaviour. Since astrocytes are central for maintaining K^+^ homeostasis, our study suggests that modulation of their inherent capabilities to clear K^+^ from the extracellular milieu is a potential mechanism to optimise neural resonance behaviour and thus tune neural oscillations.

## Introduction

Neural oscillations are rhythmic voltage fluctuations emerging from the synchronization of individual neurons that form a neuronal network. They emerge in all brain regions, and their patterns of synchrony and coherence underlie the neural code for sensory representation and short term memory^1^. The oscillations range from very slow (0.02 Hz) to fast (600 Hz) fluctuations, and are classified on the basis of power and frequency band, with different frequency bands being associated with specific behaviours^2^.

Cortical neural networks are functionally organized to enable appropriate balance of excitation and inhibition, which impacts on their synchronized activity that is fundamental for their operation. These networks are constantly alternating between different dynamic states to accommodate the large rhythmic patterns underlying the diverse cognitive functions administrated by the cortex. However, the full extent of the functional structure of these networks, especially the interactions with astrocytic networks is poorly understood.

On the local network level, neural oscillations are formed by cortical circuits that span through six layers of the cerebral cortex. The building units of these synchronous oscillations are the fluctuations in membrane potential of individual neurons known as ‘up’ (rising) and ‘down’ (falling) states. These oscillations occur both *in vitro* and *in vivo*^3^, and are routinely recorded as local field potentials^4^. Biophysical studies have revealed that single neurons are endowed with complex dynamics, including their intrinsic ability to resonate over a specific range of frequencies^5–7^. This allows them to act as resonators that respond preferentially to inputs at certain frequencies. It has been reported that many oscillatory neurons have a peak resonance frequency that is correlated with the network oscillatory activity^8,9^, and different subcellular compartments have distinct resonance properties which are also voltage dependent^10,11^.

More than a decade ago, Penttonen and Buzsáki postulated that at least ten distinct mechanisms are required to cover the large frequency range of cortical network oscillations^12,13^, and it has been reported that some frequency oscillations are driven by multiple mechanisms^14^. Several factors have been suggested to affect individual neuronal activity that underlie the generation of network oscillations, including the activation of intrinsic conductances by neuromodulators^6,13^, the influence of the dendritic structure^15^, activation of extrasynaptic receptors^16^, activation of astrocytic calcium activity^17–19^, cellular excitability^6,14^ and the hyperpolarization-activated inward current *I*_*h*_, which can modulate membrane resonance in neurons^9^ and is capable of regulating the strength and frequency of network oscillations^20^.

A recent report showed that altering the degree of excitation between different laminae of the auditory cortex can effectively switch the dominant network oscillation from granular to supragranular layers^14^. Another study showed that acetylcholine increases pyramidal cell excitability, enhances the gamma oscillations in evoked potentials and can induce theta rhythm oscillatory dynamics^6^. Thus, we hypothesise that factors that modulate ***cellular excitability*** are likely to impact network oscillatory activity.

Intrinsic cellular excitability partially depends on the reversal potential for potassium-mediated currents, as extracellular potassium concentration [K^+^]_o_ is critical in defining the resting membrane potential (RMP) of neurons and astrocytes, and is normally maintained at ~3 mM^21,22^. Sustained neuronal activity leads to local increases of [K^+^]_o_, which impact synaptic transmission and plasticity^23,24^. Hence, effective removal of K^+^ from the extracellular space is vital for maintaining physiological neuronal activity, as excessive K^+^ accumulation in the extracellular space impacts neuronal excitability and has been linked to pathological conditions^21,25,26^. For these reasons, the Sejnowski group studied the impact of [K^+^]_o_ modulation on the excitability of neurons and neuronal networks using computational models^27,28^. They found that [K^+^]_o_ dynamics can potentially modulate intrinsic conductances in neurons and thus mediate transitions between tonic spiking and bursting activity. Using their computational model, they predicted that modifications of [K^+^]_o_ can lead to alterations between fast and slow oscillatory firing modes. More recently, an *in vivo* study from Nedergaard’s group reported that neuromodulators can impact the concentration of [K^+^]_o_, regardless of synaptic activity^29^. They suggested that neuromodulators work in parallel on both neuronal spiking activity and state dependent ion homeostasis, to shift between sleep and awake states, however the exact mechanisms that govern [K^+^]_o_ were not revealed.

In the central nervous system, K^+^ homeostasis is mainly regulated by astrocytic activity, through a process termed K^+^ clearance, reviewed by^30^. Since its first proposal in 1966 by Kuffler and colleagues^31^, two major mechanisms of astrocytic K^+^ clearance have been established: *net K*^+^ *uptake*, in which the excess of [K^+^]_o_ during physiological activity is taken up by the astrocytic processes at several synapses lying within their spatial domain, and *K*^+^ *spatial buffering*, in which K^+^ ions propagate through gap-junction mediated astrocytic networks to more distal regions of the astrocytic networks^21,32^.

Physiological processes that impact neuronal depolarization can impact membrane oscillation frequency and amplitude^33^. Several studies have reported on the impact of increased [K^+^]_o_ on neuronal depolarization, however the majority of these reports were in regards to pathological conditions, such as ALS^34^, epilepsy, Rett syndrome and Huntington’s disease, reviewed by^30^. Here, we have used electrophysiological and pharmacological tools to investigate the impact of astrocytic K^+^ clearance in modulating neural oscillations at both cellular and network levels.

## Results

### High extracellular K^+^ impacts cortical oscillatory dynamics

**Figure 1:**
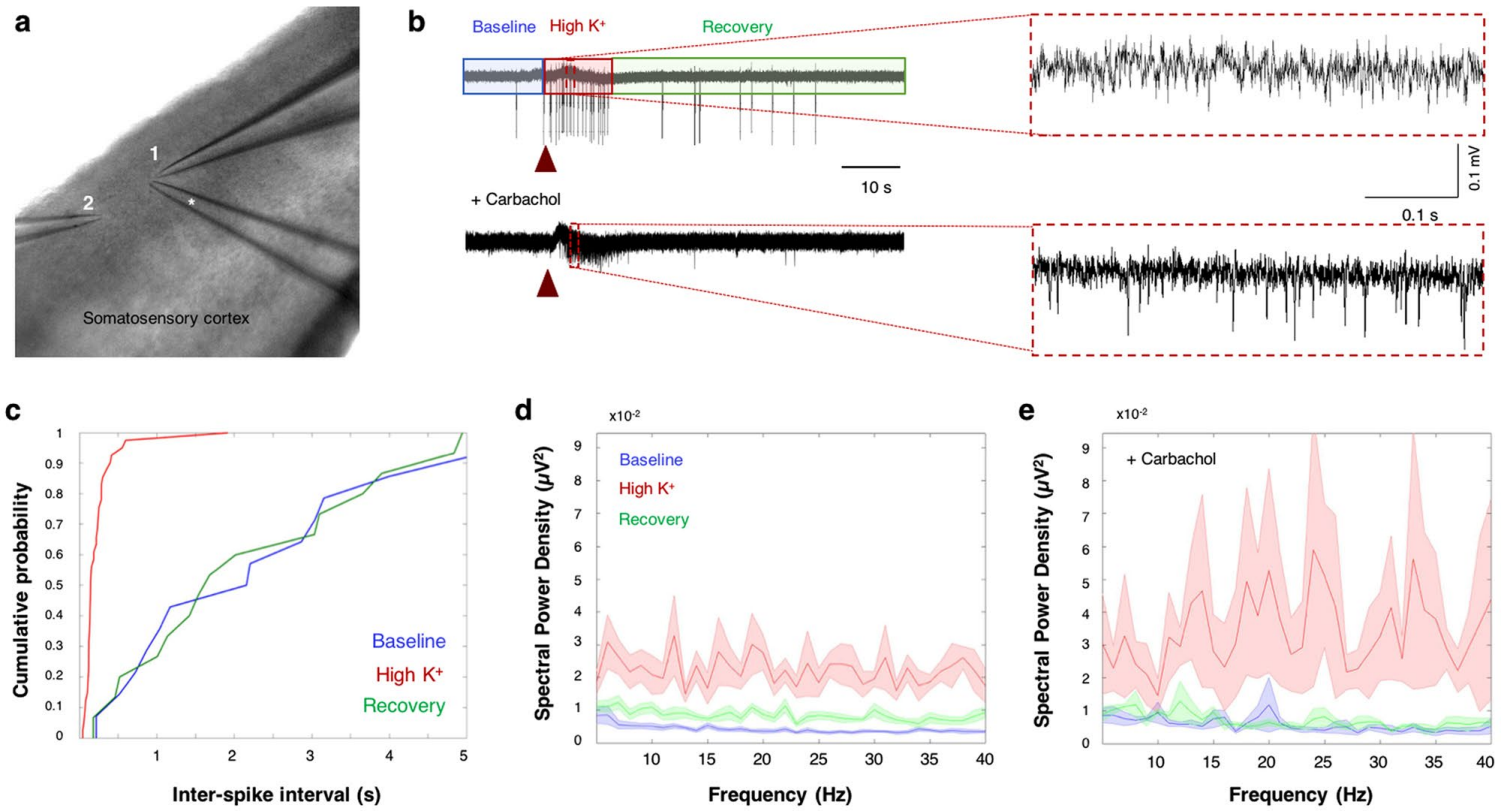
High extracellular K^+^ leads to network excitability. (**a**) Experimental setup showing the position of two recording electrodes in layer II/III of the somatosensory cortex for dual extracellular recordings. High KCl (30 mM) is locally applied to the vicinity of recording electrode 1 using a third ‘puff electrode’ (*). (**b**) Local field potentials (LFP) traces showing the network activity before and after stimulus (red arrow), both in normal aCSF (top) and in the presence of 100 μM Carbachol (bottom). Note the low-frequency (top) and high-frequency (bottom) oscillations following application of 30 mM KCl (insets). (**c**) Quantitative analysis of the inter-spike intervals during the 60-second recordings reveals a significant increase in the network spiking frequency after stimulation with 30 mM KCl (red, ‘High K^+^’) compared to ‘Baseline’ (blue) or ‘Recovery’ (green) periods under normal aCSF conditions (n = 15; KS-test p < 0.01). (**d**) Power spectrum analysis displaying the averaged (line) and standard error values (shade) of the dominating subthreshold oscillations during ‘Baseline’ (blue), ‘High K^+^’ (red) and ‘Recovery’ (green) periods, under normal aCSF (**d**) and 100 µM Carbachol (**e**). Note the power increase in multiple frequencies at both low (normal aCSF) and high (Carbachol) frequency network oscillations following stimulus.

To investigate the impact of alterations in extracellular K^+^ concentrations ([K^+^]_o_) on local network excitability and rhythmic activity, we have monitored cortical network oscillations using extracellular recordings. Two electrodes were positioned in layers II/III of the somatosensory cortex with a distance of approximately 200 µm (Fig. 1) to monitor oscillation propagation. To increase [K^+^]_o_ locally, a third electrode was used to apply high K^+^ solution (30 mM KCl) in the vicinity of the first recording electrode (Fig. 1).

Extracellular field recordings were divided into three sequential periods termed ‘***Baseline****’* - referring to non-stimulated network activity, “***High K***^***+***^” - the episode immediately following brief (1 sec) local application of KCl for defining the immediate effect of high [K^+^]_o_ (10 sec, based on multi-unit activity (MUA), see Supplementary Fig. S1), and “***Recovery***” - the recovery period during which [K^+^]_o_ was decreased by both diffusion and the K^+^ clearance process (Fig. 1). Analysis of the field recordings at each period revealed a substantial increase of both network oscillations and multi-unit (MU) activity immediately following the application of 30 mM KCl (Fig. 1b,c). While during the ‘Baseline’ period the average MU frequency was 0.13 ± 0.04 Hz (n = 15), during the ‘High K^+^’ period, MU frequency increased significantly to 2.45 ± 0.39 Hz (p < 0.01; student t-test), and returned to baseline levels during the ‘Recovery’ period (0.08 ± 0.02; Fig. 1c). Power spectrum analysis of the **network oscillations** showed that the dominant subthreshold network oscillation frequency in the ‘Baseline’ period was < 1 Hz (Supplementary Fig. S2), which is consistent with previous *in vitro* studies^3^. However, following local application of 30 mM KCl in the vicinity of the recording electrode, the oscillation power increased across a wide range of frequencies, as shown in Fig. 1d.

To evaluate the impact of increased [K^+^]_o_ on ***high frequency network oscillations***, which are usually absent in slice preparations due to limited circuitry^3^, we repeated the above experiments in the presence of the cholinergic agonist Carbachol (100 μM) that has been shown to elicit network oscillations in the gamma frequency range *in vitro*^35–37^. Our results show that bath application of Carbachol augmented the amplitude of subthreshold network oscillations at all periods (Fig. 1e). Moreover, a transient increase of [K^+^]_o_ in the presence of Carbachol led to an increase of oscillation amplitude across a wide range of frequencies, with maximum peaks at 25 & 35 Hz. These results support the concept that [K^+^]_o_ concentration can impact the network activity during both low and high frequency oscillations.

**Figure 2:**
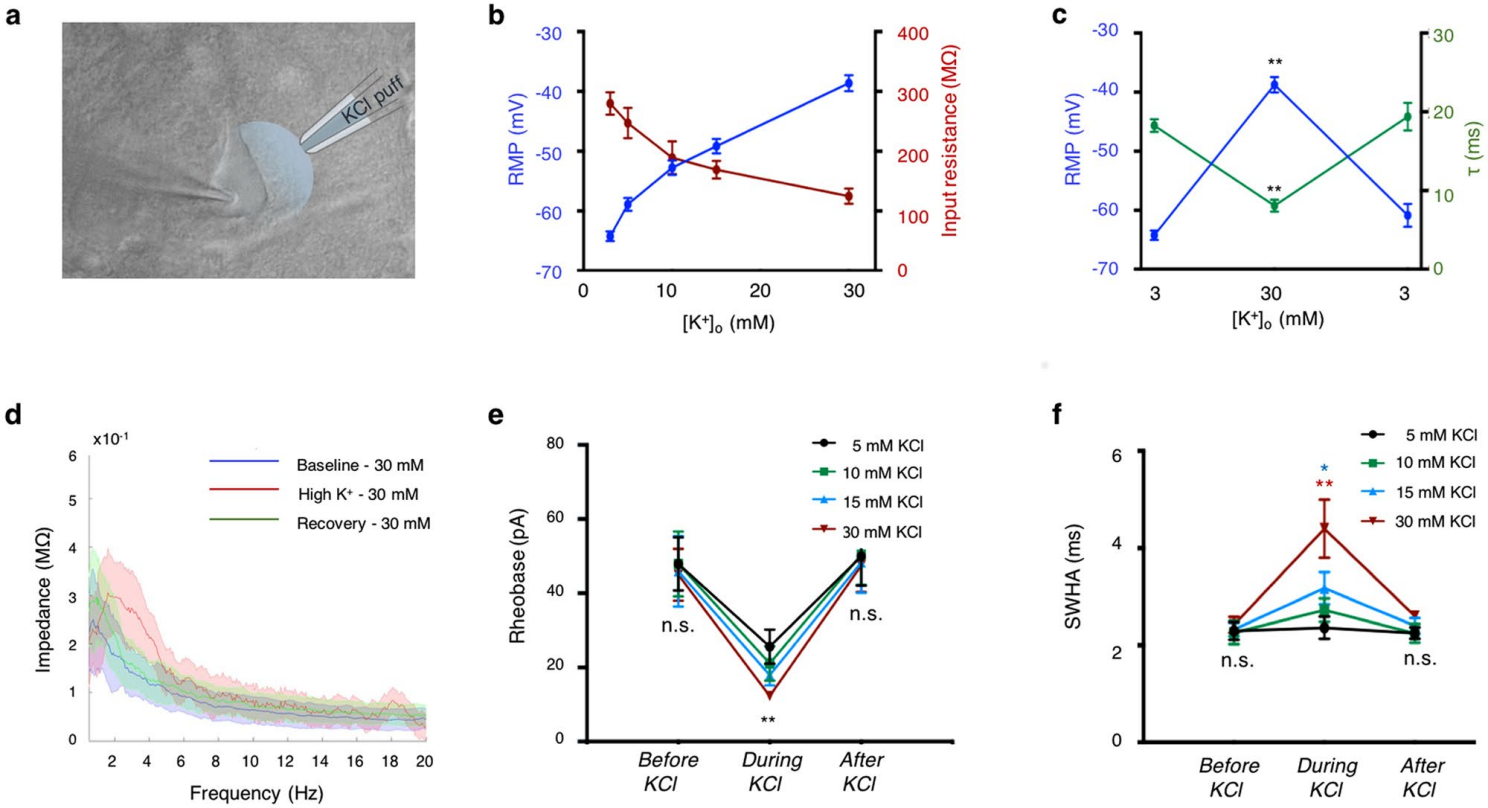
High extracellular K^+^ affects individual neuronal excitability. (**a**) DIC image showing the experimental setup. Whole-cell patch-clamp recording of layer V cortical neuron during local application of various concentrations of KCl through a puff electrode to increase the extracellular K^+^. (**b**) Elevation of [K^+^]_o_ lead to depolarization of the resting membrane potential (RMP) and decrease of the input resistance in a concentration-dependent manner. (**c**) The impact of high K^+^ on RMP and membrane time constant (τ) is transient. Note the inverse correlation of τ and RMP following increase of extracellular K^+^. (**d**) Impedance amplitude profile following ZAP protocol depicting the mean (line) and standard deviation values (shade) before, during and after local application of KCl (30 mM, color-coded) under normal aCSF conditions. Note the shift towards higher frequencies during application of elevated KCl. (**e,f**) Plots depicting the transient impact of high K^+^ on spike threshold (**e**) and spike width at half amplitude (SWHA) (**f**), in a concentration-dependent manner (30 mM n = 18; 15 mM n = 17; 10 mM n = 15; 5 mM n = 15). *P < 0.05; **P < *0.01; student t-test*.

We then studied the impact of a rise in [K^+^]_o_ on the passive and active properties of layer V cortical neurons, employing intracellular recordings (Fig. 2a). Consistent with the Goldman-Hodgkin-Katz equation for the resting membrane potential (RMP)^38^, transient bath application of KCl at different concentrations (5–30 mM) led to depolarization of the RMP of nearby neurons, in a concentration-dependent manner (Fig. 2b). The changes in membrane potential were accompanied with alterations in membrane conductance, as the membrane input resistance (R_n_) and time constant (τ) were inversely correlated to the increase in membrane potential (Fig. 2b,c; Supplementary Table S1). These alterations in membrane potential, input resistance and time constant were transient and returned to baseline values once [K^+^]_o_ was restored to 3 mM (Fig. 2c). These results are consistent with previous data showing that increased levels of [K^+^]_o_, above its physiological concentration (~3 mM), lead to significant membrane depolarization and altered synaptic function^28,39,40^.

Alterations in [K^+^]_o_ also affected membrane resonance features. Neuronal membrane resonance is determined by the interplay between active and passive membrane properties and describes the ability of neurons to respond selectively to inputs at preferred frequencies (hence resonance frequency^5^). A recent study showed that the membrane resonance frequency is strongly correlated with the network oscillation frequencies^9^. In cortical neurons, the resonance frequency has been reported to be dependent on the interplay between two currents, a slowly activating K^+^ current and a fast-persistent Na^+^ current^33^. Thus, it is not surprising that excessive K^+^ accumulation at the synaptic cleft affected the membrane resonance frequency of nearby neurons in a concentration-dependent manner. While the average peak resonance frequency at the soma was 1.5 ± 0.2 Hz at 3 mM [K^+^]_o_ (n = 14), it significantly increased to 2.5 ± 0.2 Hz at 30 mM [K^+^]_o_ (n = 12, p > 0.01; student t-test), whereas lower [K^+^]_o_ deviated the peak resonance frequency to a lesser extent (Supplementary Table S1, Supplementary Fig. S3). Moreover, following an increase in [K^+^]_o_, the full width at half amplitude (FWHA) of the impedance profile shifted significantly towards higher frequencies (3.8 ± 1.1 Hz at 30 mM [K^+^]_o_ vs 2.2 ± 0.3 Hz at 3 mM [K^+^]_o_; Fig. 2d), and restored to baseline values during the ‘Recovery’ period (2.1 ± 0.5 Hz; Fig. 2d).

Alterations of [K^+^]_o_ also affected active membrane properties, including spike rheobase and spike width at half amplitude (SWHA) in a concentration-dependent manner (Fig. 2e,f). During low [K^+^]_o_, the average spike rheobase was 47.8 ± 4.1 pA (n = 68) and decreased to 25.6 ± 4.6 pA, 21.1 ± 4.6 pA, 17.9 ± 2.8 pA, and 12.3 ± 1.0 pA following application of 5, 10, 15 and 30 mM [K^+^]_o,_ respectively (p < 0.01, student t-test, Fig. 2e, Supplementary Table S1). The average SWHA at physiological K^+^ concentrations (3 mM) was 2.3 ± 0.1 ms (n = 70) and significantly increased to 3.7 ± 0.3 ms at [K^+^]_o_ higher than 15 mM (n = 31; p < 0.01, student t-test; Fig. 2f). Once [K^+^]_o_ returned to baseline values (following washout with 3 mM K^+^ aCSF), both spike rheobase and SWHA were restored to baseline values (Fig. 2e,f).

**Figure 3:**
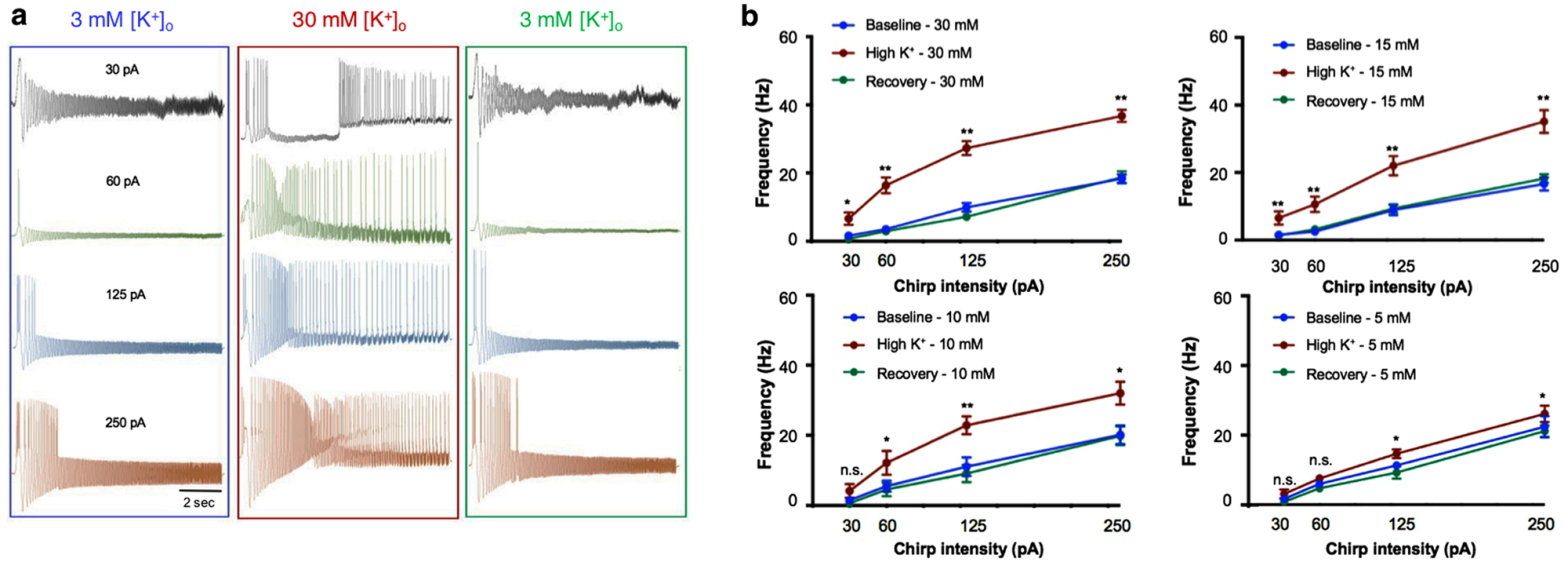
The impact of extracellular K^+^ on frequency-excitability range. (**a**) Sample traces following sinusoidal chirp stimulation (0.1–100 Hz) at different intensities (from top to bottom: 30 pA, 60 pA, 125 pA, 250 pA), recorded from the neuronal soma before (left), during (middle) and after (right) application of 30 mM KCl under normal aCSF conditions. (**b**) Frequency – excitability plot depicting the relationship between the oscillation intensity and the maximal frequency at which the cell is still excitable. Note the upward shift in the maximal frequency following application of KCl at various concentrations (30 mM KCl n = 18; 15 mM KCl n = 17; 10 mM KCl n = 15; 5 mM KCl n = 15). *P < 0.05; **P < *0.01; student t-test*.

Neuronal spiking activity underlies the execution of neuronal output and is strongly dependent on neuronal membrane resonance frequency^9^. The spike threshold is determined by a complex interaction of voltage-dependent inward and outward currents, and reflects the membrane excitability. As the membrane potential of cortical neurons is constantly oscillating, as a result of the influence of local network activity, their excitability should be investigated under similar conditions. In order to evaluate the relationship between membrane oscillation frequencies and spike threshold, we injected sinusoidal currents at different intensities (30–250 pA chirp current) in increasing frequencies (0.1–100 Hz; Fig. 3a). This protocol detects neuronal excitability at instantaneous sinusoidal frequencies^41^, and allows an evaluation of the relationship between neuronal excitability and oscillatory behaviour (Fig. 3a), depicted by the Frequency-Spiking plot (Fig. 3b).

Our results indicate that a transient increase of [K^+^]_o_ led to a shift of the frequency excitability range towards higher frequencies, in a concentration-dependent manner across different stimulus intensities (Fig. 3b). Under baseline conditions (3 mM of [K^+^]_o_), the maximal frequency in which layer V cortical neurons were excitable was 18.9 ± 1.1 Hz (250 pA, n = 43), and increased to 36.8 ± 1.8 Hz once local [K^+^]_o_ increased to 30 mM. The frequency excitability range returned to previous values, once [K^+^]_o_ returned to baseline (18.7 ± 1.7 Hz; Fig. 3b). These results emphasize the impact of a rise in [K^+^]_o_ on neuronal excitability at both cellular and network levels, supporting an active role for K^+^ homeostasis in modulating network activity.

### Modulation of astrocytic K^+^ clearance impacts neuronal excitability and network rhythmicity

Astrocytic K^+^ clearance following neuronal activity is mediated via several transporting mechanisms, commencing with K^+^ uptake via Kir4.1 channels and Na^+^/K^+^-ATPase, through distribution of K^+^ across astrocytic gap junctions (Cx30/Cx43) to astrocytes with lower K^+^ concentration, and terminated by redistribution of K^+^ to distant cortical areas^30^. To study the dynamic role of *astrocytic K*^+^ *clearance* mechanisms in modulating network oscillations, we have measured the local network activity while modifying either K^+^ uptake through Kir4.1 channels that are selectively expressed in astrocytes and are responsible for ~45% of the K^+^ uptake^32,42,43^, or K^+^ distribution through the astrocytic syncytium via selective blockade of Cx43 gap junctions.

### Modulation of K^+^ uptake by astrocytes

**Figure 4:**
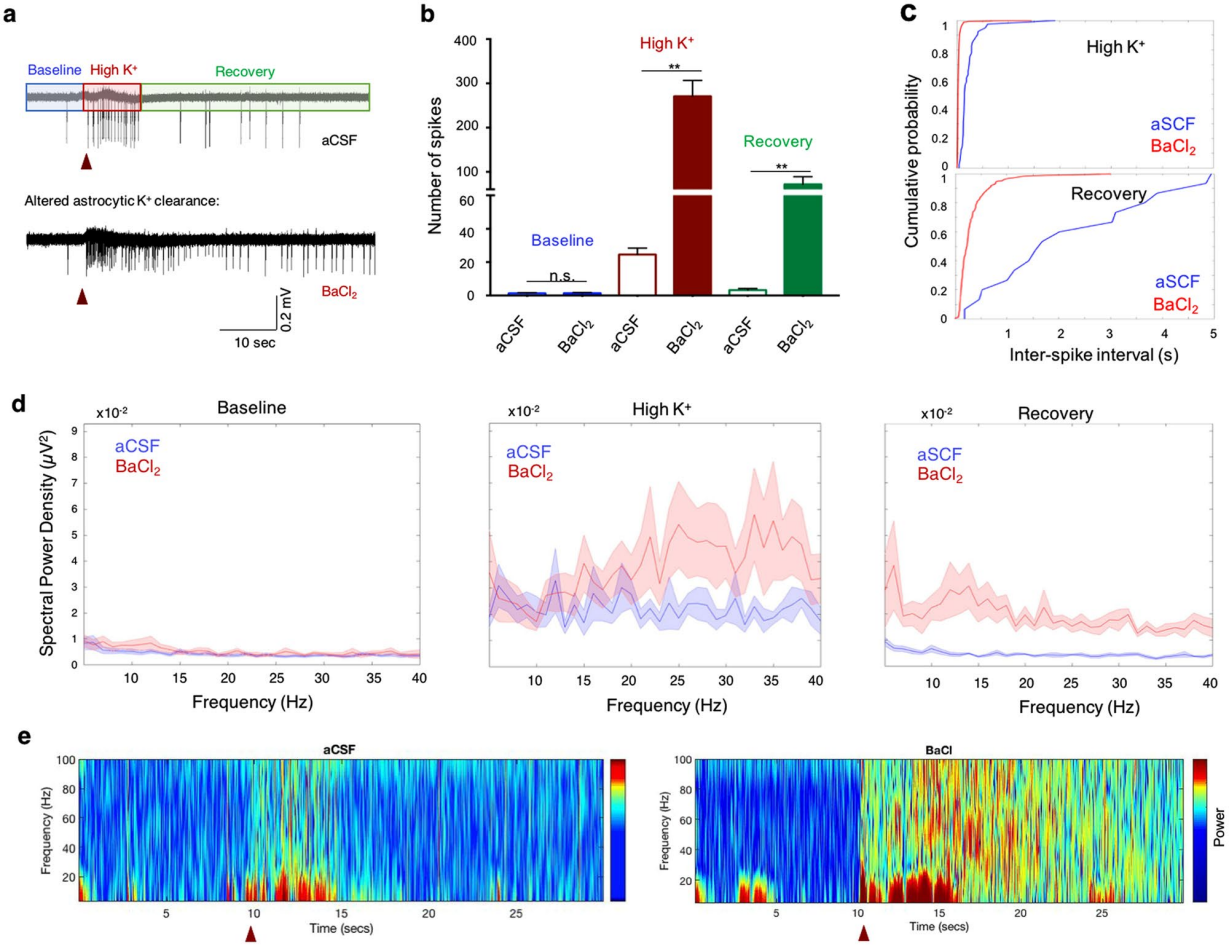
Modulation of astrocytic K^+^ uptake affects network excitability. (**a**) Extracellular recordings showing the network activity before and after stimulus with 30 mM KCl (red arrow), in normal aCSF (top) and after bath application of 100 μM BaCl_2_ (bottom). The network spiking activity has been divided into three periods for subsequent analysis: ‘Baseline’, ‘High K^+^’ and ‘Recovery’ periods. (**b**) Quantitative analysis of the network excitability during the different episodes (color-coded) revealed an increase in the number of spikes following stimulus (High K^+^), which decrease during the “Recovery” period in normal aCSF (n = 15). Blocking K^+^ uptake via bath application of BaCl_2_ results in a significant increase in the number of spikes (**b**) and decrease in inter-spike intervals (**c**) during both ‘High K^+^’ and ‘Recovery’ periods (n = 16; p < 0.01; KS-test p < 0.01). (**d**) Power spectrum analysis depicting the averaged (line) and standard error values (shade) of the dominant oscillation frequencies governing Baseline, High K^+^ and Recovery periods under normal aCSF and reduced astrocytic K^+^ clearance (BaCl_2_, top) conditions. Note the increase in the oscillation power at frequencies in the beta and gamma range under 100 μM BaCl_2_. (**e**) Colour coded spectrogram of the network oscillations in normal aCSF (left) and following bath application of 100 μM BaCl_2_ (right). Red triangles at the bottom indicate the time of local application of 30 mM KCl. *P < 0.05; **P < *0.01; student t-test*.

Barium is a non-specific K^+^ channel inhibitor, but concentrations up to 100 µM predominantly inhibit the Kir subfamily^44^, whereas higher concentrations affect the Na^+^/K^+^-ATPase (reviewed by^32^). Bath application of BaCl_2_ at low concentration (100 µM) did not affect the network activity during the baseline period (Fig. 4a), as cortical slices usually have low spiking activity in the absence of stimulation^3^. However, following local application of KCl (30 mM), inhibition of astrocytic Kir channels led to a substantial increase in network excitability, expressed as a significant rise of the MU frequency (27.1 ± 3.6 vs 2.45 ± 0.4, n = 16; p < 0.01; student t-test; Fig. 4b) and reduction of the inter-spike intervals (BaCl_2_ vs ACSF; p < 0.01, KS-test, Fig. 4c). Similarly, analysis of the ‘Recovery’ period, during which K^+^ is cleared from the extracellular milieu, showed that compared to normal aCSF, blockade of K^+^ uptake by astrocytes led to a significant increase of the MU frequency (1.8 ± 0.4 Hz vs 0.08 ± 0.02 Hz, p < 0.01, student t-test; Fig. 4b) and reduction in the inter-spike intervals (p < 0.01, KS-Test; Fig. 4c), indicating network hyperexcitability. Moreover, Barium significantly increased the duration of the ‘Recovery’ period compared to normal aCSF (Supplementary Fig. S1, aCSF vs BaCl_2_, p < 0.01), indicating that modulation of astrocytic Kir4.1 channel activity, which affects the removal of excessive [K^+^]_o_, can extend the hyper-synchronous firing activity at the network level.

**Figure 5:**
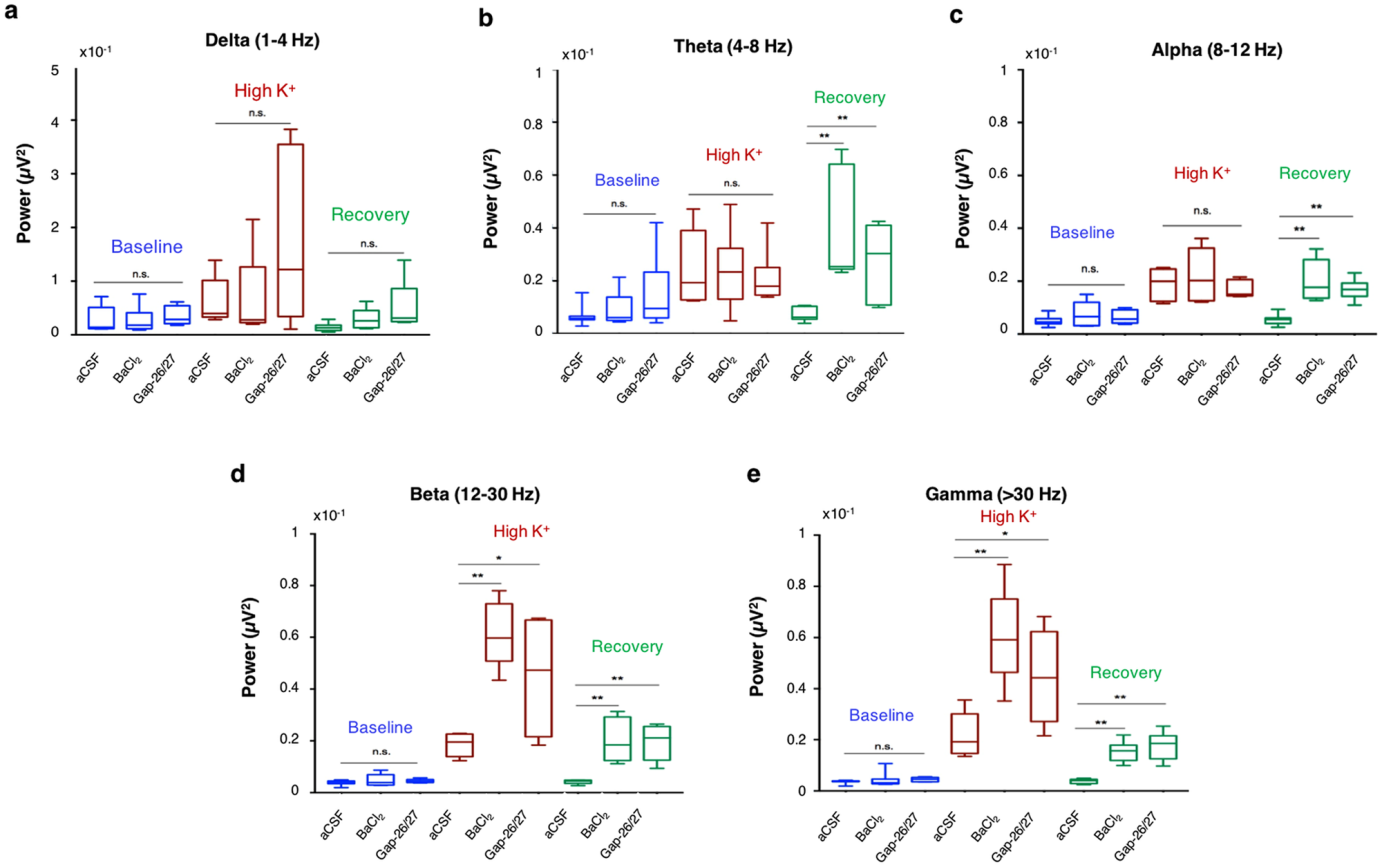
Modulation of astrocytic K^+^ clearance increase the power of cortical oscillations at multiple frequencies. (**a**) Power spectrum density of cortical oscillations were divided into five frequency bands: delta (1–4 Hz), theta (4–8 Hz), alpha (8–12 Hz), beta (12–30 Hz) and gamma (>30 Hz) (**a–e**). The average power for each trial was than plotted during ‘Baseline’, ‘High K^+^’ and ‘Recovery’ periods. Comparison of the average power during the ‘High K^+^’ period revealed that modulation of astrocytic K^+^ clearance by BaCl_2_ or Gap-26/27 lead to significant rise at the Beta and Gamma range (**d**,**e**). Comparison of the average power during the ‘Recovery’ period show a significant rise in the theta, alpha, beta and gamma frequencies, once astrocytic K^+^ clearance is impaired. *P < 0.05; **P < *0.01; student t-test*.

Analysis of the field potential during Barium application showed that the power of cortical oscillations increases significantly across multiple frequencies at both ***‘High K***^***+***^***’*** and ‘***Recovery***’ periods (Fig. 4d). To compare between the spectrum densities of network oscillations, we divided the power spectrum into five frequency bands: delta (1–4 Hz), theta (4–8 Hz), alpha (8–12 Hz), beta (12–30 Hz) and gamma ( > 30 Hz). Our results indicate that blockade of Kir4.1 channels led to an increase of the oscillation amplitude across different frequencies, peaking in the beta and gamma range during the ‘High K^+^’ period (Fig. 4d; Fig. 5d,e), and theta and beta range during the ‘Recovery’ period (Figs 4d, and 5). These results indicate that high frequency network oscillations are supported by enhanced network excitability, mediated by a significant rise of [K^+^]_o_.

### Modulation of K^+^ distribution via the astrocytic syncytium

**Figure 6:**
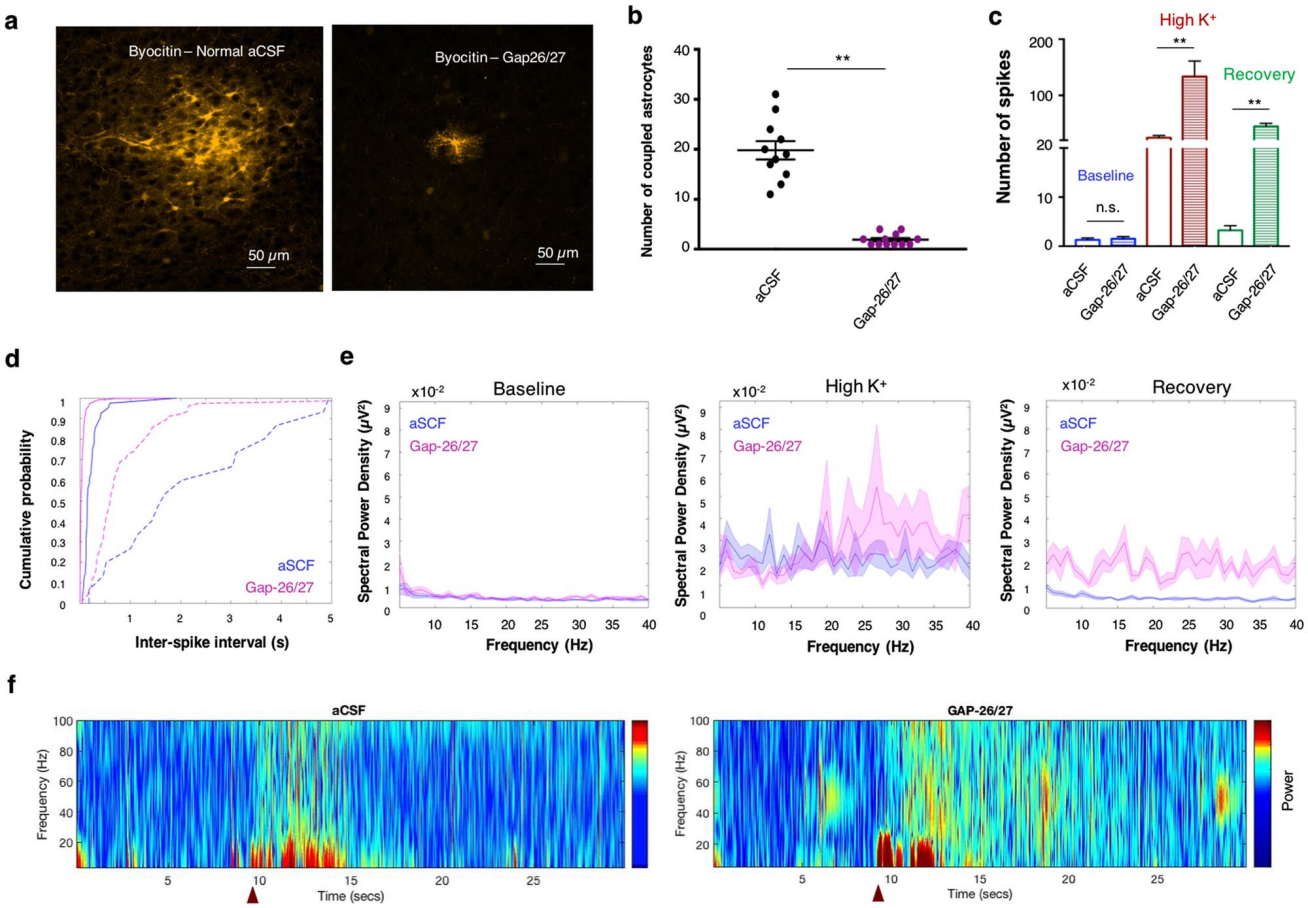
Reduced astrocytic connectivity impacts on network excitability and oscillation frequencies. (**a**) 40x confocal image of biocytin-stained astrocytes in layer II/III of the somatosensory cortex, depicting the astrocytic network under normal aCSF conditions (left) and following application of GAP-26/27 (right). (**b**) Selective blockade of connexin 43 by pretreatment of GAP26/27 mixture significantly decreases astrocytic connectivity compared to normal aCSF conditions (n = 12, p < 0.01). (**c**) Quantitative analysis of the network excitability at the different periods (color-coded) reveals that reduction in astrocytic connectivity leads to a significant increase in the number of spikes during both “High K^+^” and “Recovery” periods compared to normal aCSF (n = 13, p < 0.01). (**d**) Cumulative probability distribution of inter-spike intervals during “High K^+^” (continuous line) and “Recovery” (dashed line) periods is shifted towards the left in the presence of Gap-26/27 compared to normal aCSF (n = 13, KS-test p < 0.01). (**e**) Power spectrum analysis depicting the averaged (line) and standard error values (shade) of the dominant subthreshold oscillation frequencies governing Baseline, High K^+^ and Recovery periods under normal aCSF and Gap-26/27 conditions. Note the increase in the oscillation power at frequencies in the beta and gamma range. (**f**) Colour coded spectrogram of the network oscillations in normal aCSF (left) and following bath application of GAP-26/27 (right). Red triangles at the bottom indicate the time of local application of 30 mM KCl. **P < *0.01; student t-test*.

We next assessed the impact of K^+^ clearance via the astrocytic syncytium on the network activity. To selectively disrupt the astrocytic syncytium activity, we incubated the slices with a mixture of connexin 43 mimetic peptides (GAP-26, 200μM and GAP-27, 300μM), that selectively decrease astrocytic connectivity via electrical gap junctions (Fig. 6a,b), as previously reported by^45,46^. This led to a decrease in astrocytic coupling, as indicated by a significant decline of directly connected astrocytes from 19.8 ± 1.8 to 1.9 ± 0.3 (n = 12, p < 0.01; Fig. 5b; see also Supplementary Fig. S4).

Following modulation of astrocytic connectivity, we observed a significant increase of neuronal excitability during the ‘High K^+^’ and ‘Recovery’ periods, expressed as an increase in MU frequency (13.36 ± 2.73 Hz vs 2.45 ± 0.39 Hz at ‘High K^+^’, and 1.12 ± 0.13 vs 0.08 ± 0.02 at ‘Recovery’ period; n = 13; p < 0.01, student t-test; Fig. 6c) and reduction in the inter-spike intervals (p < 0.01, KS-test; Fig. 6d). Moreover, the oscillation power increased across a wide range of frequencies, especially at the beta and gamma range during ‘High K^+^’ (Figs 6e and 5), and most frequencies during the ‘Recovery’ period, indicating that modulation of the processes that affect the removal of excessive [K^+^]_o_ result in hyperexcitable activity at the network level.

### Alterations of astrocytic K^+^ clearance modulate the oscillatory properties of neurons

**Figure 7:**
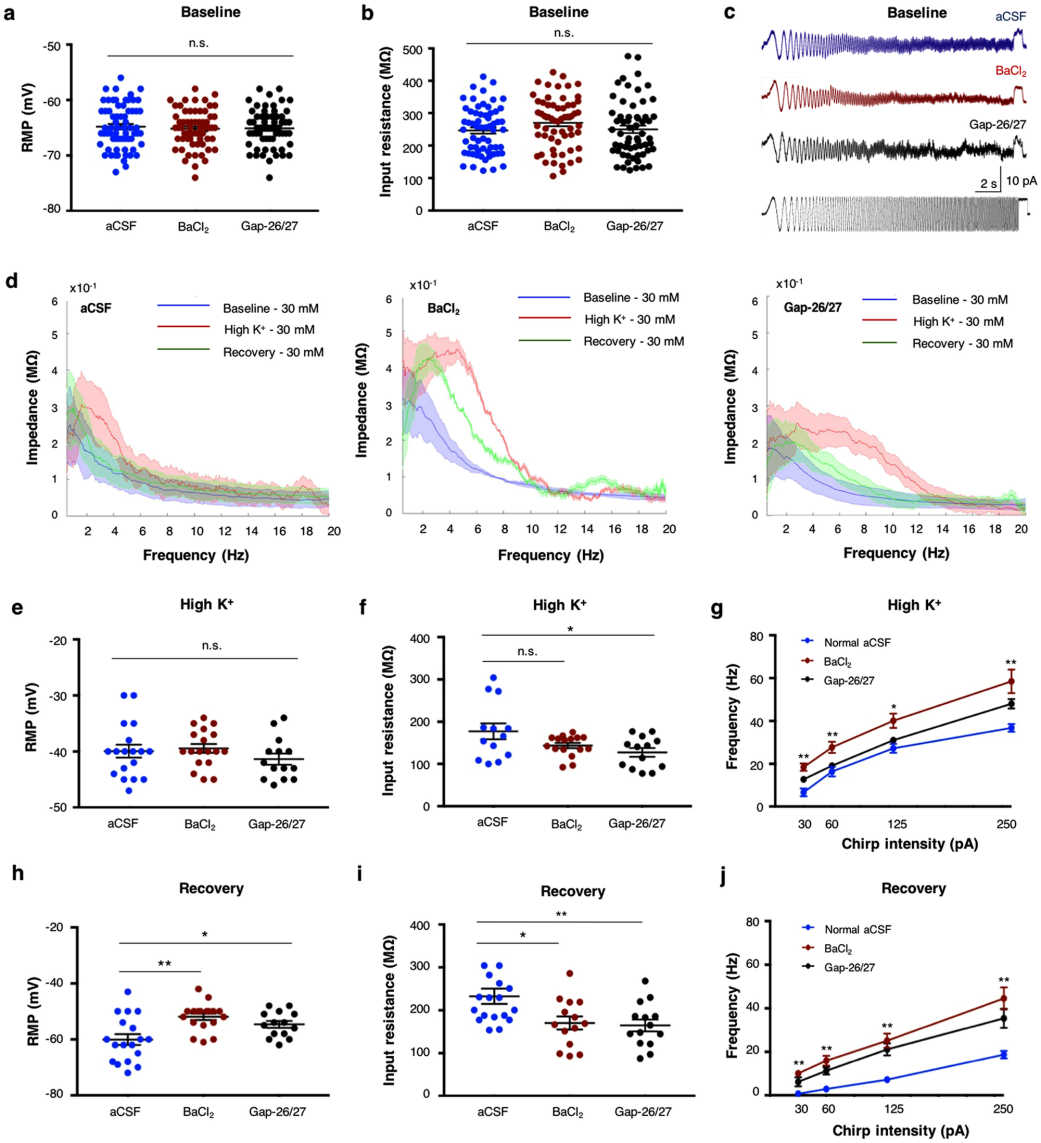
Modulation of astrocytic K^+^ clearance impacts on membrane properties of nearby neurons. (**a**) Comparison of the membrane properties between normal aCSF (n = 66), BaCl_2_ (n = 63) and Gap-26/27 (n = 60) conditions, depict no significant differences in RMP (**a**) or input resistance (**b**) during the ‘Baseline’ period. (**c**) Sample traces of membrane oscillations recorded during sinusoidal subthreshold ZAP stimulation (10 pA, bottom trace) under normal aCSF (top), BaCl_2_ (middle) and Gap-26/27 (bottom) conditions at the ‘Baseline’ period. (**d**) Impedance frequency profiles depicting the averaged (line) and standard deviation (shade) values of the resonance frequency recorded at the soma before, during and after application of 30 mM KCl (color-coded), under normal aCSF (left), 100 μM BaCl_2_ (middle) and GAP-26/27 (right) conditions. (**e**) Comparison of the membrane properties between normal aCSF (n = 18), BaCl_2_ (n = 18) and Gap-26/27 (n = 14) conditions at the ‘High K^+^’ period, depict no alterations in RMP (**e**) and decrease in input resistance following application of Gap-26/27 (**f**); p < 0.05). (**g**) Plot of the frequency-excitability range during the ‘High K^+^’ period depicts a significant increase in the maximum frequency under BaCl_2_ (n = 18) and GAP 26/27 (n = 14) compared to normal aCSF (n = 18). (**h**) Alterations in astrocytic K^+^ clearance result in a significant depolarization of the RMP (**h**) and decrease of the input resistance (**i**), which led to an upward shift of the frequency-excitability range (**j**). *P < 0.05; **P < *0.01; student t-test*.

Evaluation of the impact of local alterations in astrocytic K^+^ uptake and buffering on nearby layer V cortical neurons revealed a substantial influence on both passive and active membrane properties. While in the ‘Baseline’ period, all membrane properties were comparable, either following bath application of BaCl_2_ (n = 63) or GAP-26/27 mixture ((n = 60; Fig. 7a–c), during the ‘High K^+’^ period, the membrane resonance peaked at higher frequencies (BaCl_2 =_ 4.4 ± 0.2 Hz, n = 12; GAP 26/27 = 4.1 ± 0.3 Hz, n = 13; p < 0.01 student t-test; Fig. 7d), and the impedance profile indicated a widening of the FWHA from 3.8 ± 1.1 Hz to 6.7 ± 0.8 Hz with BaCl_2_, and to 8.0 ± 1.3 Hz with Gap26/27, suggesting that the membrane can resonate over a wider range of frequencies once astrocytic clearance is impaired. These alterations in the impedance profile were more noticeable in the ‘Recovery’ period, in which the FWHA increased from 2.1 ± 0.5 Hz to 4.8 ± 0.6 with BaCl_2_, and 5.2 ± 1.1 Hz following incubation with Gap26/27 (Fig. 7d; Supplementary Table S1).

These deviations in membrane resonance were accompanied with a decrease of the input resistance during both ‘High K^+^’ and ‘Recovery’ periods (Fig. 7f,i) and depolarization of the resting membrane potential during the ‘Recovery’ period (Fig. 7h), consistent with the results of high [K^+^]_o_ (Fig. 2b). Moreover, modulation of astrocytic K^+^ clearance led to firing of action potentials at higher frequencies as indicated by an upward shift of the frequency spiking range at both ‘High K^+^’ (Fig. 7g) and ‘Recovery’ periods (Fig. 7j).

## Discussion

Network rhythmic oscillations are a product of complex neural activity in networks of multiple neurons that are activated synchronously. This synchronous activity of oscillating networks is viewed as the critical link between single-neuron activity and behaviour, or ‘brain state’, and as such, it is a diagnostic tool used both clinically and in brain research, reviewed by^2^. In a recent paper, Lee & Dan suggested that there are two fundamental questions concerning brain states: i) what mechanisms control brain states and ii) what is the function of each state^47^. We suggest that astrocytes play an essential role in the mechanisms that gear the transition between the different brain states.

The transition between distinctive network oscillation frequencies is due to recruitment of discrete neural networks, which are associated with different behavioural tasks, such as sleep, learning, and attention^2,48,49^. Animals constantly switch between behavioural states, and thus oscillation frequencies, as a reaction to the ever-changing environment. Regulation of the transition between these oscillations is essential for animal survival, and disruption of the normal regulatory mechanisms results in disorders such as epilepsy, Rett syndrome and sleep apnoea, reviewed by^30^. However much remains to be learned about the exact mechanisms that modulate this transition.

Our results indicate that alteration of [K^+^]_o_ modulates network excitability, affecting both low and high frequency oscillations (Figs 1, 5). At the cellular level, enhanced excitability of individual neurons is seen by the transient decrease in the rheobase, accompanied by alterations in membrane properties including decrease of input resistance, reduced time constant, depolarization of the resting membrane potential, and shift of the oscillatory properties towards higher frequencies (Fig. 2), that result in an upward shift of the frequency range in which neurons were still excitable (Fig. 3). These results are consistent with experimental *in vivo*, *in vitro*, and modelling studies of transitions between cortical active and silent states, showing that periods of high activity are accompanied by a decrease in input resistance mediated by a simultaneous increase in both excitation and inhibition^28,50,51^, as well as increase in voltage-gated conductances as a consequence of membrane depolarization^52^. At the network level, enhanced excitability is depicted as increased MU frequency and increase in oscillation power over a wide spectrum of frequencies (Figs 1, 4).

Subthreshold membrane potential fluctuations of individual neurons are strongly correlated with their local network activity^53,54^, which is also influenced by their resonance frequency^2^. The resonance frequency is an intrinsic property of the neuron, emerging from the effects of the membrane leak conductance and capacitance, however it can be modulated by voltage dependent currents^5,33^. Previous reports showed that network oscillations are dependent on K^+^ dynamics^55^, however the exact mechanism was not resolved. Here we show for the first time that modulation of astrocytic K^+^ clearance can impact the resonance frequency of single neurons (Fig. 7d), promoting their oscillatory activity over a wider frequency spectrum (Figs 7g,j), that impacts the network oscillatory behaviour (Figs 4d and 6d).

For proper function, astrocytes can function either individually within their spatial domain^56^, or as a syncytium, reviewed by^30^. K^+^ clearance from the extracellular milieu is a multi-step process that requires firstly K^+^ taken up by individual astrocytic processes, and then recruiting of the astrocytic network to transfer the excess K^+^ ions to regions with low K^+^ concentration. We have divided the extracellular recordings into three periods that are correlated to the different stages of the astrocytic clearance process. During the ‘Baseline’ period, [K^+^]_o_ concentration is low (3 mM), obviating the need for clearing mechanisms. The ‘High K^+^’ period is characterized by an immediate local increase in [K^+^]_o_ as occurs following repetitive spiking activity, or release of neuromodulators^29^, which slowly decreases due to activation of K^+^ uptake by astrocytes, as well as diffusion to distant regions. Although we refer to this time window as a single period, it is not homogenous, as the [K^+^]_o_ concentration and thus uptake mechanism reduces with time. The recovery period characterizes the processes taking place while high [K^+^]_o_ is washing out, and local [K^+^]_o_ is slowly decreasing to baseline values. During this period, [K^+^]_o_ is mainly decreased via astrocytic uptake and less through diffusion.

Blocking astrocytic K^+^ uptake with a low concentration of Barium (BaCl_2_, 100 μM) enhanced the resonance frequency range of individual neurons at both ‘High K^+^’ and ‘Recovery’ periods (Fig. 7, Supplementary Fig. S3), which led to a significant increase of the maximal spiking frequency (Fig. 7) and MU frequency (Fig. 4, Supplementary Fig. S1). At the network oscillations level, blocking Kir channels results in augmentation of the oscillation power at the beta and gamma frequency range during the ‘High K^+^’ period, and most frequencies during the ‘Recovery’ period (Figs 4, 5).

Consistent with these results, incubation of cortical slices with a mixture of Cx-43 antagonists (GAP-26, GAP-27), that interrupt the astrocytic coupling and thus stopped them from working as a syncytium (Fig. 6), increased the duration of the ‘Recovery’ period (Supplementary Fig. S1). Moreover, blocking astrocytic coupling boosted the maximal spiking frequency at both ‘High K^+^’ and ‘Recovery’ periods (Fig. 6), and enhanced the resonance frequency range (Fig. 7, Supplementary Fig. S3) that led to enhancement of the oscillation power in the beta and gamma range during the ‘High K^+^’ period. Furthermore, disruption of the astrocytic coupling augmented power oscillations across most frequencies during the ‘Recovery’ period (Figs 5, 6), emphasizing the importance of astrocytic coupling in mediating K^+^ homeostasis and facilitating the transition between network oscillations. However, blocking gap junctions can also effect other processes that require astrocytic coupling for their physiological activity, including calcium waves, which might affect the power of these cortical oscillations.

In his seminal work, Steriade showed that the network oscillation frequencies are inversely correlated to the oscillations power, and that fast oscillations correspond to desynchronized states, while slow frequency-high amplitude oscillations correspond to synchronised network activity^48^. The recruitment of neurons into specific networks that control each brain state has been attributed to the activity of neuromodulators such as acetylcholine (ACh), noradrenaline (NA), serotonin (5-HT), dopamine (DA), and histamine (HA), originating from distinct groups of neurons located in subcortical areas^47,57,58^. While agonists of these neuromodulators affects arousal and attention, leading to desynchronization of cortical activity^47,58,59^, their antagonists promote sleep and synchronous network activity. Yet, despite their crucial role in brain function, it remains unclear how these neuromodulators coordinate state-dependent, global changes in neuronal activity.

Recently Ma and colleagues showed that neuromodulators can signal through astrocytes, by affecting their calcium oscillations, to alter neuronal network activity^60^. Consistent with their work, Nedergaard’s group showed that neuromodulators can impact the concentration of extracellular K^+^, regardless of synaptic activity^29^, and suggested that neuromodulators work in parallel on both neurons and astrocytes, to maximise their impact on synchronous activity and recruitment of neurons into networks. Our results depicting the astrocytic impact on [K^+^]_o_ dynamics and network oscillatory behaviour are consistent with this view. We show that modulation of different phases in the clearance process, either at the uptake level or buffering through the astrocytic network, results in alterations of the oscillatory activity of individual neurons, as well as the network behaviour.

## Materials and Methods

### Animals

We used P21-P28 days-old mice expressing GFP under a GFAP promoter (strain 003257, Jax laboratories). All animals were healthy and handled with standard conditions of temperature, humidity, twelve hours light/dark cycle, free access to food and water, and without any intended stress stimuli. All experiments were approved and performed in accordance with Western Sydney University committee for animal use and care guidelines (Animal Research Authority #A10588).

### Slice preparation

Animals were deeply anesthetized by inhalation of isoflurane (5%), decapitated, and their brains were quickly removed and placed into ice-cold physiological solution (artificial CSF, aCSF) containing (in mM): 125 NaCl, 2.5 KCl, 1 MgCl_2_, 1.25 NaH_2_PO_4_, 2 CaCl_2_, 25 NaHCO_3_, 25 glucose and saturated with carbogen (95% O_2_−5% CO_2_ mixture; pH 7.4). Parasagittal brain slices (300 μm thick) were cut with a vibrating microtome (Leica VT1200S) and transferred to the Braincubator^TM^ (PaYo Scientific, Sydney), as reported previously^61^. The Braincubator is an incubation system that closely monitors and controls pH, carbogen flow and temperature, as well as irradiating bacteria through a separate UV chamber^62,63^. Slices were initially incubated for 12 min at 35 °C, after which they were allowed to cool to 15–16 °C and kept in the Braincubator^TM^ for at least 30 min before any measurement^64^.

### Electrophysiological recording and stimulation

The recording chamber was mounted on an Olympus BX-51 microscope equipped with IR/DIC optics and Polygon 400 patterned illuminator (Mightex). Following the incubation period in the Braincubator, slices were mounted in the recording chamber for a minimum of 15 min, to allow them to warm up to room temperature (~22 °C), and were constantly perfused at a rate of 2–3 ml/min with carbogenated aCSF, as reported previously^65^.

*Extracellular recordings* of network oscillations were performed by placing two recording electrodes, tip diameter of 1 μm (2–3 MΩ), in layer II/III of the somatosensory cortex, with an inter-electrode distance of one barrel (appx 200 µm). Network oscillations were induced by reducing the concentration of Ca^2+^ (1.2 mM) and Mg^2+^ (1 mM) in the bath solution, as previously reported^3^. Transient increase of extracellular K^+^ was achieved by applying a 1-second positive pressure (∼0.2 ml) of potassium chloride (KCl) at different concentration through a puffing electrode (tip diameter of 2 μm, ∼1 MΩ) placed in the vicinity of the 1^st^ recording electrode (Fig. 1).

*Whole-cell intracellular recordings* from layer V pyramidal neurons in the somatosensory cortex were obtained with patch pipettes (5–7 MΩ) containing (in mM): 130 K-Methansulfate, 10 HEPES, 0.05 EGTA, 7 KCl, 0.5 Na_2_GTP, 2 Na_2_ATP, 2 MgATP, 7 phosphocreatine, and titrated with KOH to pH 7.2 (∼285 mOsm). Voltages were recorded in current clamp mode using a multiclamp 700B dual patch-clamp amplifier (Molecular Devices), digitally sampled at 30–50 kHz, filtered at 10 kHz, and analysed off-line using pClamp 10 software^41^. Cells were considered stable and suitable for analysis if the input resistance did not change more than 20% during the baseline recordings, before any treatment.

Membrane properties were obtained before, during and after local application of KCl at different concentrations (30, 15, 10, 5 mM), applied through a puffing electrode placed in the vicinity of the recording electrode. Following application of KCl, the puffing electrode was removed to allow potassium wash-out for 2 minutes before resuming intracellular recordings.

### Suprathreshold sinusoidal stimulus protocol

In order to evaluate the alterations in suprathreshold oscillation frequencies under different conditions, 10-second stimulating protocols of sinusoidal current (chirp stimulation), in which there was a linear increase in the frequency from 0.1 to 100 Hz, were designed at 30, 60, 125 and 250 pA, using the pClamp 10 software suit (Molecular devices, Sunnyvale, CA) and injected to the neuronal soma through the recording electrode.

### Measuring astrocytic coupling

To evaluate the degree of astrocytic coupling, individual astrocytes were loaded with intracellular solution containing biocytin (0.3%; Sigma) for 12 min in whole cell mode (supplementary Fig. S4). After a 12-min labelling period, the slices were immersed in fixative (4% paraformaldehyde in phosphate-buffered saline (PBS), pH 7.4) at 4°C for at least 24 h and then stored in PBS. To visualize the biocytin-filled cells, the slices were treated with 1:200 Alexa-Fluo conjugated Streptavidin in PBS with 1% Triton X-100 for 48 h, followed by washing in PBS, as previously described by^66^.

To inhibit gap junction coupling in astrocytes, slices were pre-treated with a mixture of GAP-26 (200 μM, AnaSpec) and GAP-27 (300 μM, AnaSpec) for 15 min before patch clamp recordings. Stained astrocytes were counted using the “pointpicker” plugin of ImageJ. The average intensity of the Biocytin signals was used as cut-off to distinguish the recorded cells from other cells that were coupled to it. For each slice, z-stacks were typically acquired to include the entire biocytin-stained syncytia.

#### Power spectral density

To assess the dominant frequencies of the network oscillations under different conditions we used power spectrum density (PSD) analysis. The principal frequencies were disclosed by applying Fast Fourier Transform (FFT) directly to the raw data, as previously described by^67^. Each recording was divided into three distinct sections: ‘Baseline’, ‘High K^+^’, and ‘Recovery’, based on inter-spike interval observations under normal conditions, as described in supplementary Fig. S1. As there are multiple trials of each experiment, the PSD was calculated for each period using an appropriately sized Hamming window and the DC component was discarded. The power spectrum was binned into 1 Hz frequency bands, from which the mean and the standard error of the power were extracted and averaged for all trials. Only the first 40 Hz of the power spectrum is examined, and the results shown using the same y-axis limits to allow for comparison.

#### Spectrum analysis

To measure the resonance frequency of individual neurons, a 20-second subthreshold sinusoidal current at 10 pA, with a linear increase in frequency from 0.1 to 20 Hz (chirp stimulation) was applied through the recording electrode, as previously described^41^. The resonance frequency was determined as the peak in the impedance amplitude profile (ZAP) generated by dividing the Fourier transforms of the voltage signal by that of the current signal, as previously described by^33^. The voltage recordings were recorded in millivolts and the current signal recorded in picoamps and adjusted accordingly such that the resulting complex impedance can be measured in Ohms. The DC component of each FFT was discarded by removing the first element of each FFT signal, and only the real component of the resulting impedance was considered.

To better assess the effect of the interventions, the Full Width at Half Maximum (FWHM) was calculated for the impedance of each recording in each condition. The baseline impedance was removed from the signal by subtracting the mean impedance in the 18 Hz to 20 Hz frequency band prior to calculating the FWHM. The first five impedance samples of each recording were also discarded due to variations in the DC offsets in the signals used to calculate the impedance. The FWHM was calculated by normalizing the signal to a range of [-0.5, 0.5] and finding the distance in the x-axis between the first two zero crossings.

To visualize the changes in oscillation strength over time, we have plotted the data via spectrogram. The spectrogram is the decomposition of the extracellular signal into its spectral components (power by frequency) across time^68^. The target period for this analysis was 10 sec before KCl application and 20 sec after KCl application.

### Drugs

All drugs were stored in frozen stock solutions and were added to aCSF just before recordings. Some experiments were performed in the presence of Carbachol (100 μM, Sigma Aldrich) to elicit high frequency oscillations, or BaCl_2_ (100 μM, Sigma Aldrich) to block astrocytic Kir4.1 channels, in the bath solution. To assess the role of Cx43-composed gap junctions, brain slices were incubated with GAP-26 (200 μM, AnaSpec) and GAP-27 (300 μM, AnaSpec) mixture for 15 minutes and then transferred to the recording chamber for electrophysiological recordings.

### Statistical analysis

Unless stated, data is reported as mean ± S.E.M. Statistical comparisons were done with Prism (GraphPad Software; San Diego, CA) using two-tailed unpaired student t-test and one-way ANOVA, according to the experimental design. Power spectrum and resonance frequency analyses were performed using Matlab (Mathworks). Probability values < 0.05 were considered statistically significant.

### Data availability statement

All data generated or analysed during this study are included in this published article (and its Supplementary Information files).

## Electronic supplementary material

Dataset1
