# Supplementary material for: Astrocytic modulation of cortical oscillations
Source: Sci Rep. 2018 Aug 1;8:11565. doi: 10.1038/s41598-018-30003-w (PMC6070488; doi:10.1038/s41598-018-30003-w)
Supplement: Supplementary file 1 — Dataset1 [file 41598_2018_30003_MOESM1_ESM.pdf]

Astrocytic modulation of cortical oscillations

Alba Bellot-Saez[1, 2], Greg Cohen[1], André van Schaik[1], Lezanne Ooi[3], John Morley[1, 2] and Yossi Buskila[1,2*]

[1] Biomedical Engineering and Neuroscience group, The MARCS Institute, Western Sydney University, Penrith, NSW, Australia

[2] School of Medicine, Western Sydney University, Campbelltown, NSW, Australia

[3] Illawarra Health and Medical Research Institute, School of Biological Sciences, University of Wollongong, NSW, Australia

* Correspondence: Y.buskila@Westernsydney.edu.au

Bldg 30, Goldsmith Ave, Campbelltown, NSW 2560, AUSTRALIA

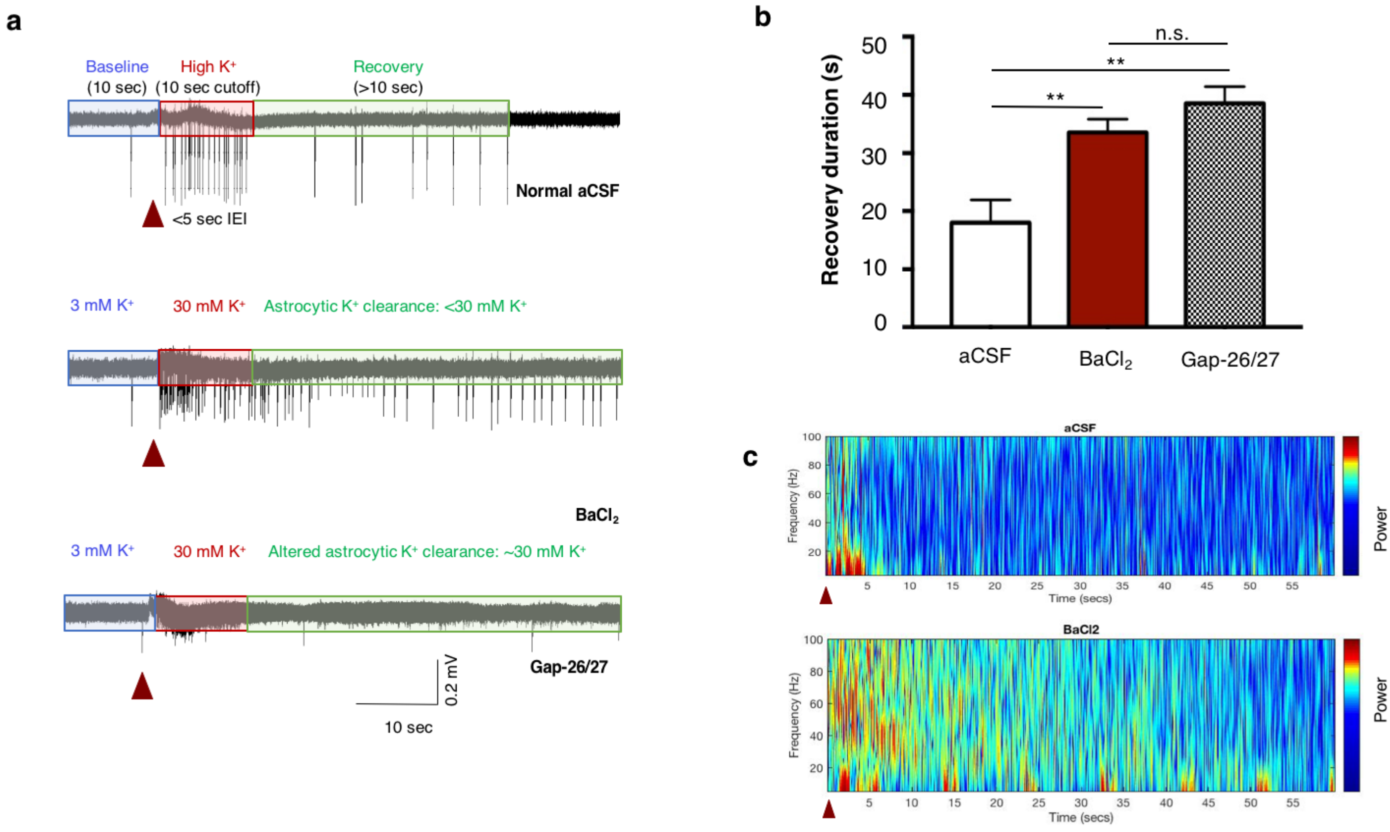

Supplementary Figure S1. Measuring network excitability. a) Traces of extracellular recordings showing the network activity before and after brief (1 sec) application of 30 mM KCl (red arrow), in normal aCSF (3 mM K^+, top) and after bath application of 100 µM $BaCl_2$ (middle) or Gap-26/27 (bottom). For subsequent analysis, the network activity was divided into three episodes: ‘Baseline’ - the period prior to stimulation; ‘High K^+’, the immediate period following application of 30 mM KCl. The duration of the ‘High K^+’ period was set to 10 sec as this was the average time in which the inter-spike intervals was <5 sec (3 mM K^+ aCSF, n=15). The third period was termed ‘Recovery’, as this is the period where $[K^+]_o$ decreases to baseline levels due to diffusion and astrocytic K^+ clearance. The duration of the Recovery period was set as the period where the inter-spike intervals were <10 seconds. b) Bar graph depicting the impact of impairment of K^+ uptake ($BaCl_2$, n=16) and gap junction blockers (Gap-26/27, n=13) on the duration of the Recovery period. c) The power spectrogram of extracellular oscillations following KCl application (time 0, red triangles) depicting the increase in power at higher frequencies during the “High K^+” (first 10 sec) and “recovery” (from 10 sec onwards) periods.

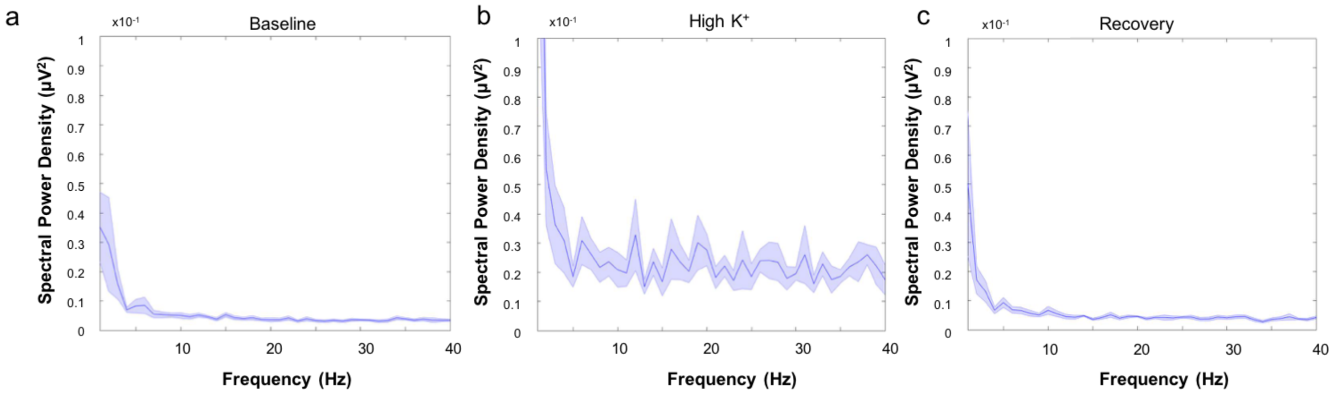

Supplementary Figure S2. Power spectrum analysis of low frequency network oscillations. Power spectrum analysis depicting the averaged (line) and standard error values (shade) of the dominant oscillation frequencies governing Baseline (a), High K^+ (b) and Recovery (c) periods depicting maximal power at oscillation frequencies <1 Hz under normal aCSF conditions.

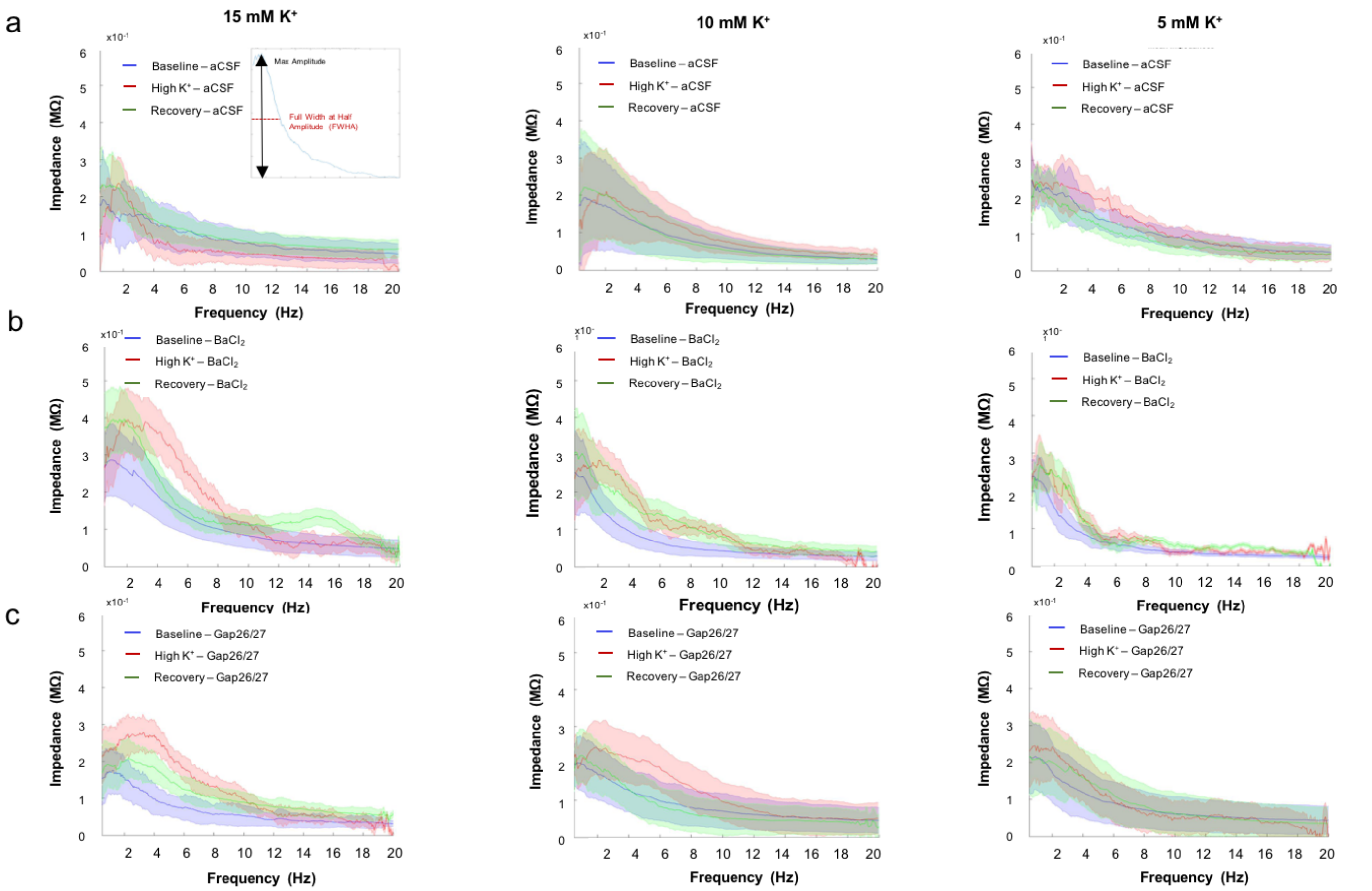

Supplementary Figure S3. The impact of $[K^+]_o$ on the resonance frequency. a) Impedance amplitude profile depicting the mean (line) and standard deviation values (shade) of the maximal voltage response to a subthreshold chirp stimulation (10 pA), before, during and after (color-coded) stimulation at different concentrations of KCl (5 -15 mM) under normal aCSF conditions. Note the shift towards higher frequencies during the 'High K^+' period. Inset - schematic representation of the Full Width at Half Amplitude (FWHA) calculated by Matlab. The function first measures the maximal amplitude of the resonance frequency curve from baseline values at ~20 Hz to maximal peak, and then measures the full width of the maximum amplitude. b,c) ZAP profile under $BaCl_2$ (b) and Gap-26/27 (c) conditions. Note the shift towards higher frequencies during 'High K^+' and 'recovery' periods compared to normal ACSF.

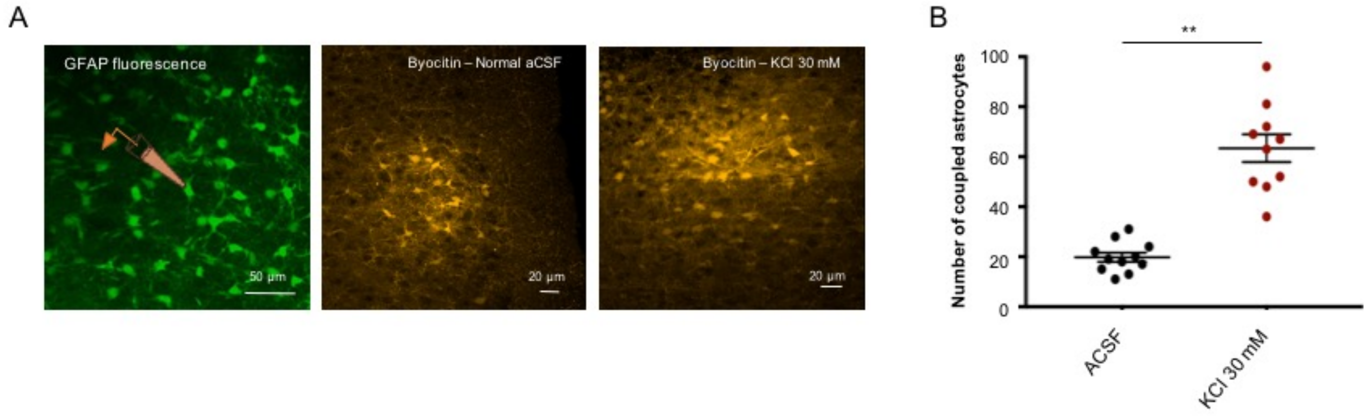

Supplementary Figure S4. High $[K^+]_o$ enhances astrocytic coupling in the cortex. a) Measuring astrocytic coupling through Biocytin labeling. Left – image of an astrocyte from GFAP-GFP mouse filled with Biocytin through the recording electrode (12 min) to determine its direct connections with surrounding astrocytes. Right – Confocal images (20x objective) showing the biocytin-stained astrocytic network in layer II/III of the somatosensory cortex from GFAP mouse under normal aCSF (middle), and following application of 30 mM KCl (right). b) Local application of 30 mM KCl significantly increases the degree of coupled astrocytes in layer II/III of the cortex. *$^{**}P<0.01$; student t-test.*

$[K^+]_o$ (mM)		RMP (mV)	*R*in (MΩ)	Rheobase (pA)	Spike width (ms)	f_R (Hz)	FWHA (Hz)
**3**	**ACSF**	**-65.1±0.5 (n=66)**	**248.9±10.5 (n=65)**	**47.8±4.1 (n=68)**	**2.3±0.1 (n=70)**	**1.4±0.1 (n=51)**	**2.1±0.3 (n=51)**
**5**	Before KCl puff	-65.8±1.2 (n=13)	264.7±22.5 (n=14)	46.2±6.8 (n=14)	2.3±0.2 (n=15)	1.4±0.2 (n=12)	2.2±0.6 (n=12)
	During KCl puff	-57.9±1.4 (n=13)	247.0±25.4 (n=14)	25.6±4.6 (n=13)	2.4±0.2 (n=14)	2.0±032 (n=12)	2.6±0.8 (n=12)
	After KCl puff	-65.3±1.8 (n=12)	257.0±30.1 (n=13)	47.5±7.2 (n=13)	2.3±0.1 (n=14)	1.3±0.2 (n=11)	2.3±0.7 (n=11)
**10**	Before KCl puff	-65.1±1.7 (n=13)	255.9±29.4 (n=12)	47.9±8.7 (n=14)	2.2±0.2 (n=15)	1.3±0.3 (n=12)	2.4±0.8 (n=12)
	During KCl puff	-52.8±1.2 (n=13)	189.2±27.2 (n=12)	21.1±4.6 (n=13)	2.7±0.2 (n=13)	2.0±0.2 (n=10)	2.7±0.4 (n=10)
	After KCl puff	-64.1±1.8 (n=12)	240.7±36.5 (n=12)	48.3±8.2 (n=13)	2.3±0.2 (n=13)	1.3±0.2 (n=12)	2.2±1.1 (n=12)
**15**	Before KCl puff	-64.4±0.9 (n=17)	232.8±18.8 (n=17)	47.3±10.1 (n=17)	2.3±0.1 (n=17)	1.5±0.2 (n=13)	1.8±0.9 (n=13)
	During KCl puff	-49.2±1.2 (n=17)	169.1±14.8 (n=16)	17.9±2.8 (n=16)	3.2±0.3 (n=16)	2.1±0.3 (n=13)	2.9±1.0 (n=13)
	After KCl puff	-64.2±1.8 (n=16)	223.9±25.0 (n=16)	50.4±8.1 (n=16)	2.4±0.2 (n=16)	1.2±0.1 (n=13)	1.9±0.4 (n=13)
**30**	Before KCl puff	-65.0±1.0 (n=18)	247.2±23.2 (n=17)	49.7±7.1 (n=18)	2.4±0.2 (n=18)	1.5±0.2 (n=14)	1.9±0.2 (n=14)
	During KCl puff	-38.6±1.3 (n=18)	125.5±14.4 (n=16)	12.3±1.0 (n=16)	4.4±0.6 (n=15)	2.5±0.2 (n=12)	3.8±1.1 (n=12)
	After KCl puff	-60.9±1.9 (n=18)	43.0±5.9 (n=16)	50.0±7.9 (n=16)	2.6±0.1 (n=15)	1.3±0.2 (n=11)	2.1±0.5 (n=11)

Supplementary Table S1. The impact of extracellular K^+ on the biophysiological properties of layer V cortical neurons.

**Data is reported as mean ± S.E.M (n = number of neurons recorded).*
